# Metastatic BRAF V600E-Mutated Adenocarcinoma of the Lung Presenting as Extreme Neutrophilia and Eosinophilia

**DOI:** 10.7759/cureus.18930

**Published:** 2021-10-20

**Authors:** Azharuddin Muhammad, Hari K Nair, Sunil Tulpule, Adnan Khan

**Affiliations:** 1 Hematology and Medical Oncology, Saint Louis University School of Medicine, Saint Louis, USA; 2 Internal Medicine, Saint Louis University School of Medicine, Saint Louis, USA

**Keywords:** paraneoplastic leukemoid reaction, metastatic non-small cell lung cancer, braf v600e mutations, eosinophilia, neutrophilia

## Abstract

Hematologic paraneoplastic syndromes with extreme neutrophilia and eosinophilia are very rarely associated with adenocarcinoma of the lung. We describe a case of a 57-year-old female who presented with neutrophil- and eosinophil-predominant hyperleukocytosis and hypoxic respiratory insufficiency. Bone marrow biopsy confirmed metastatic adenocarcinoma, similar to the biopsy-proven adenocarcinoma of the lung. She was administered one dose of cytotoxic chemotherapy with carboplatin and pemetrexed and started on leukoreductive therapy with hydroxyurea. Molecular testing revealed a BRAF V600E mutation and she was started on dabrafenib and trametinib with significant clinical improvement. This is the first reported case of metastatic BRAF V600E mutated non-small cell lung cancer presenting with extreme neutrophilia and eosinophilia treated with a combination BRAF and mitogen-activated extracellular kinase (MEK) inhibitor.

## Introduction

Cancers of the lung and bronchus accounted for 12.4% of all new cancer cases and 21.7% of all cancer deaths in 2021 [1]. Lung cancers have been associated with mild leukocytosis [2,3]. BRAF mutations are found in 1-4% of non-small cell lung cancer (NSCLC) cases and are rarely seen with marked neutrophilia and eosinophilia [4].

Malignancy‐induced paraneoplastic leukemoid reaction (PLR) is difficult to diagnose since several secondary causes need to be ruled out [3]. If the underlying malignancy is not clinically apparent, PLR can be mistaken for myeloproliferative neoplasms, thereby altering the patient’s management [5].

Other than myeloproliferative neoplasm (MPN), there are many other etiologies that can cause leukemoid reaction, including infections (e.g., clostridium difficile colitis, disseminated tuberculosis, severe shigellosis), malignancies (e.g., carcinomas, Hodgkin's lymphoma, melanoma, sarcoma), drugs (e.g., corticosteroids, minocycline, recombinant hematopoietic growth factors), intoxication (e.g., ethylene glycol), or severe hemorrhage or acute hemolysis [6].

This is the first reported case of a patient with BRAF V600E-mutated metastatic adenocarcinoma of the lung who presented with extreme neutrophilia and eosinophilia as a hematologic paraneoplastic syndrome and is being treated with dabrafenib and trametinib.

## Case presentation

A 57-year-old female with a medical history of Hashimoto thyroiditis presented to an urgent care center with three months of progressive dyspnea and dry cough. Chest x-ray demonstrated bilateral consolidations consistent with pneumonia. She was treated with a course of doxycycline without clinical improvement. Repeat chest x-ray demonstrated bilateral pulmonary nodules and she underwent bronchoscopy with bronchoalveolar lavage that reported non-specific pneumonitis with intra-alveolar hemorrhage and capillaritis. Computed tomography (CT)-guided biopsy of right pulmonary nodule showed poorly differentiated lung adenocarcinoma immuno-histochemical stains were positive for cytokeratin (CK) 7 and negative for CK20 and negative for thyroid transcription factor 1 (TTF-1) and P40. Complete blood count (CBC) with differential showed marked neutrophilia and eosinophilia suggestive of bone marrow invasion by malignancy. Bone marrow biopsy revealed a hypercellular marrow with metastatic carcinoma similar to the adenocarcinoma seen from the pulmonary nodule biopsy involving 15% core cellularity.

**Figure 1:**
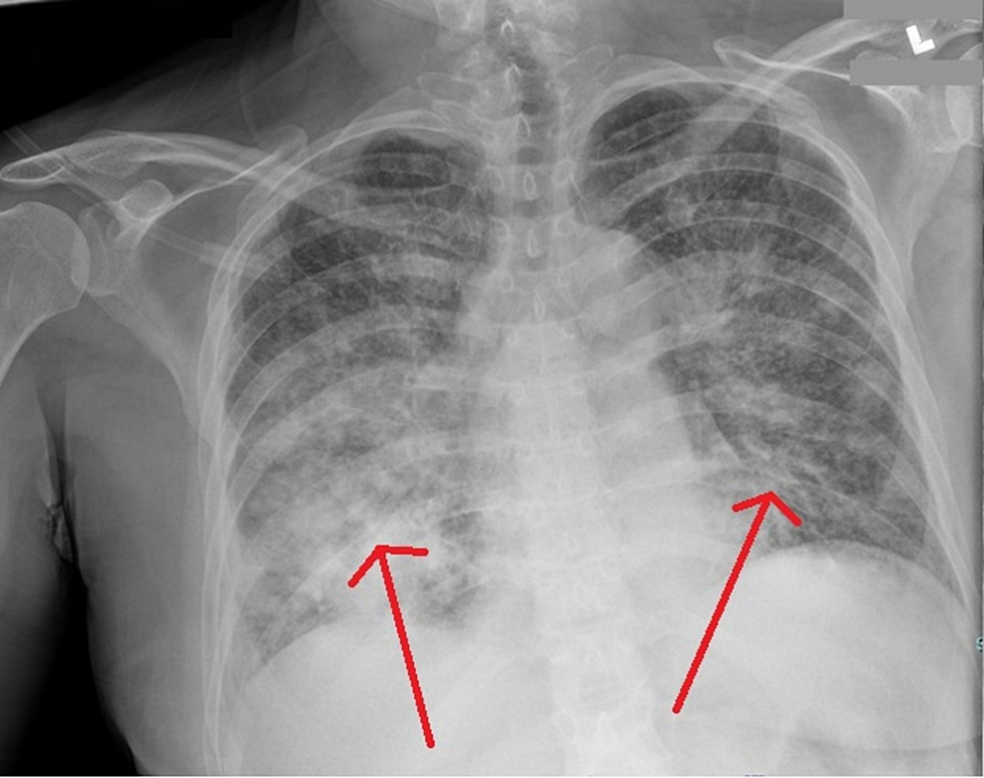
Chest X-ray Portable Anteroposterior (AP) Arrows show bilateral consolidation and nodular opacities, right greater than left, which may represent some combination of confluent metastatic disease, pneumonia, and pulmonary edema. The heart size is normal. Mediastinal fullness is concerning for adenopathy. There is a small right pleural effusion. No left pleural effusion. No pneumothorax.

**Figure 2:**
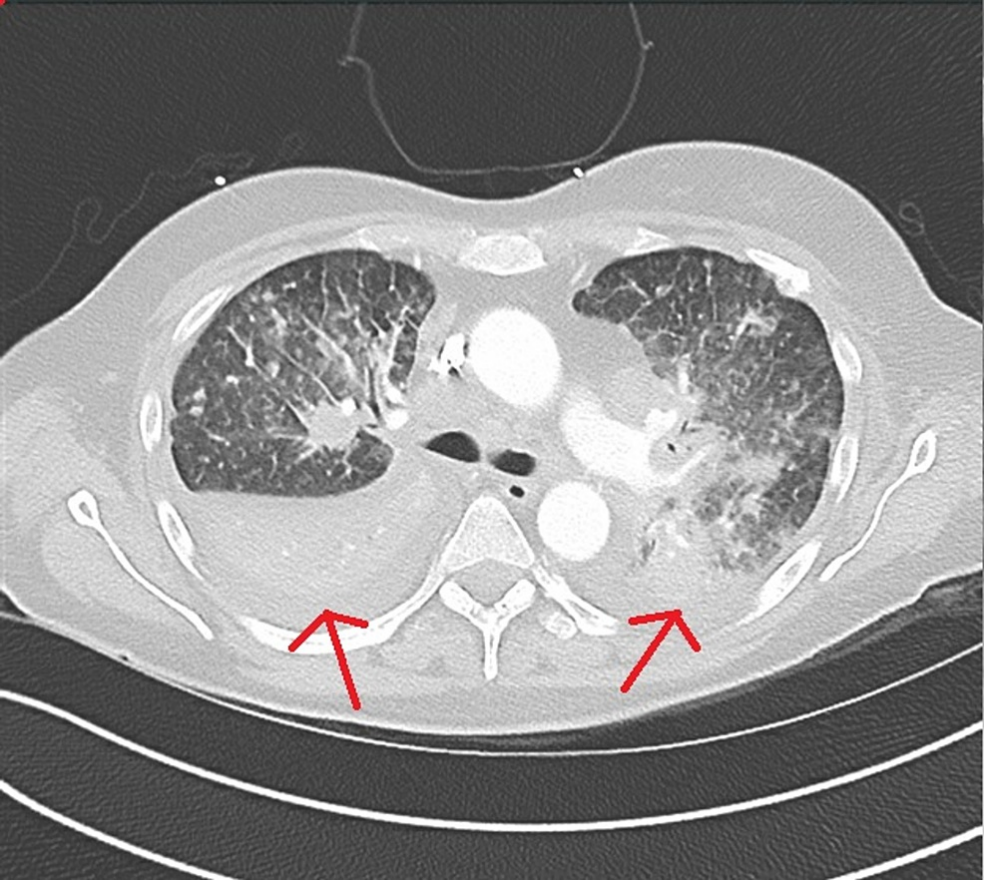
CT angiogram of the chest No evidence of acute pulmonary embolism. Innumerable pulmonary nodules with underlying lymphangitic carcinomatosis in the bilateral lungs. Bilateral mediastinal and hilar lymphadenopathy. Likely represent metastatic disease. Arrows showing moderate bilateral pleural effusions persist with atelectasis/consolidation of the right lower lobe, minimally increased since the prior examination.

Chest x-ray demonstrated extensive bilateral airspace disease (Figure 1). Transthoracic echocardiogram revealed normal left ventricular systolic function and filling pressures with no wall motion abnormalities. CT angiogram of the chest demonstrated progression of mediastinal and hilar lymphadenopathy with innumerable bilateral pulmonary nodules consistent with metastatic disease (Figure 2). She subsequently developed hypoxic respiratory insufficiency requiring hospitalization.

**Figure 3:**
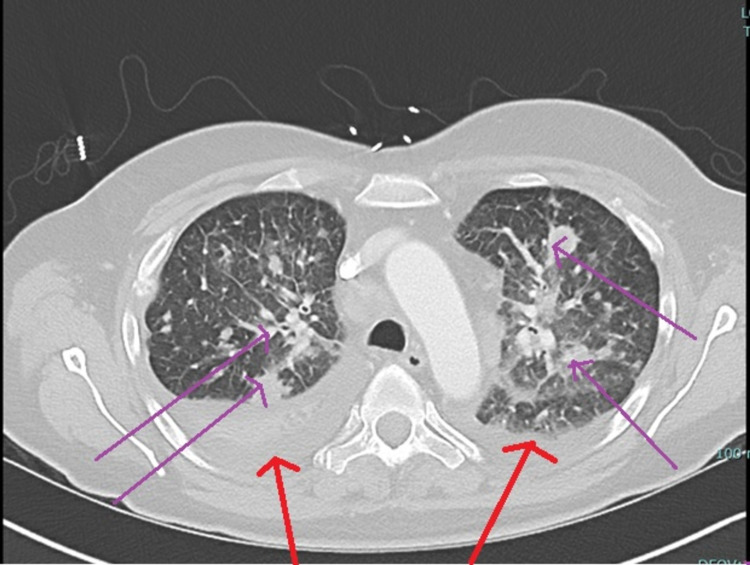
CT of chest, abdomen, and pelvis The purple arrow shows innumerable pulmonary masses and lymphangitic carcinomatosis in bilateral lungs with focal areas of hypoattenuation consistent with necrosis and mediastinal and bilateral hilar lymphadenopathy with mass effect on multiple bronchi. This is consistent with the patient's biopsy-proven metastatic adenocarcinoma. The red arrow shows bilateral pleural effusions and compressive atelectasis of the right lower lobe.

**Figure 4:**
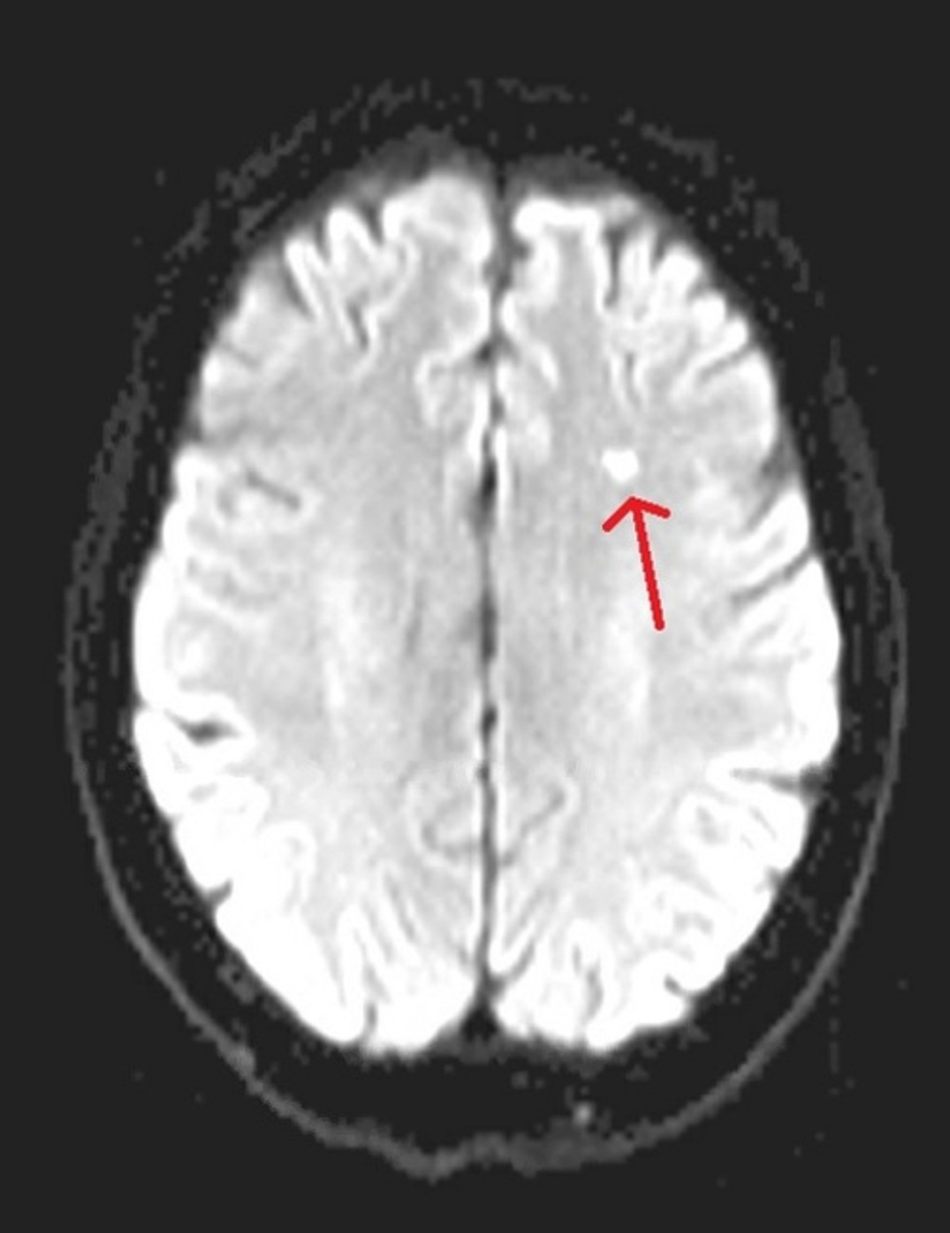
MRI of the brain Arrow depicts ischemic infarct in the left frontal lobe.

On presentation, she was tachypneic, dyspneic, and hypoxic requiring supplemental oxygen via nasal cannula. Labs showed marked leukocytosis to 116 x 10^3^ cells/mcL in peripheral blood with absolute neutrophil count of 55,780 cells/mcL and absolute eosinophil count of 51,130 cells/mcL. Hemoglobin and platelet count were normal at 15.2 g/dL and 284 x 10^3^ cells/mcL, respectively. CT of chest, abdomen, and pelvis with contrast was performed to rule out concomitant infection and disease progression - which demonstrated innumerable pulmonary masses, lymphangitic carcinomatosis in bilateral lungs, mediastinal and bilateral hilar lymphadenopathy, bilateral pleural effusion, hypoattenuating foci in the liver and spleen, and left adrenal nodule and prominent mesenteric lymph nodes - was suspicious for extensive metastatic disease (Figure 3). Magnetic resonance imaging (MRI) of the brain was done to rule out brain metastasis, which demonstrated three small ischemic infarctions and bone marrow-replacing lesion in the right-parietal bone (Figure 4).

Due to her high burden of disease and clinical deterioration, she received one dose of palliative systemic chemotherapy with intravenous carboplatin area under the curve (AUC) 5 mg/mL/min and pemetrexed 500 mg/m^2^. Immune therapy was not administered because molecular studies were pending at the time. 

She had a progressive rise in her total leukocyte count to 137 x 10^3^ cells/mcL with an increased absolute eosinophil count of 78,600 cells/mcL and absolute neutrophil count of 57,920 cells/mcL. Her respiratory status worsened with increased supplemental oxygen requirements to a high-flow nasal cannula with a fraction of inspired oxygen (FiO2) of 80%. She was initiated on steroid therapy with dexamethasone 20 mg orally twice daily which was then switched to prednisone 60 mg orally daily, with the subsequent addition of hydroxyurea 1000 mg orally twice daily for cytoreduction.

Programmed death-ligand 1 (PD-L1) tumor proportion score (TPS) was 80% and she was noted to have a BRAF V600E mutation. She was transitioned from chemotherapy to targeted therapy with dabrafenib, a mutated B-raf protein kinase inhibitor, and trametinib, a reversible and selective inhibitor of the mitogen-activated extracellular kinase (MEK). 

After eight days of targeted therapy, there was a significant improvement in her respiratory status and normalization of total leukocyte count to 6.3 x 10^3^ cells/mcL with normalized absolute eosinophilia to 210 cells/mcL and absolute neutrophilia to 1820 cells/mcL. She was weaned off oxygen support to room air and was discharged home. Four months later, the patient remains on dabrefenib and trametinib with stable disease.

## Discussion

Tumor-induced leukocytosis can be mistaken for infection or myeloproliferative neoplasms (MPN) if the underlying malignancy is not clinically evident [6]. Infection should initially be excluded since it is more common than PLR as a cause of secondary leukocytosis. Other causes of secondary leukocytosis include tuberculosis and *Clostridium difficile*, drugs such as corticosteroids, ethylene glycol intoxication, acute hemolysis, and miscellaneous etiologies [3,6]. In patients who have PLR, granulocyte-colony stimulating factor (G-CSF) is directly secreted by tumor cells into the host’s circulation, thus leading to cytokine-mediated granulocytosis [6].

Since PLR can present similarly to MPNs, pathologists usually differentiate in peripheral blood smear or a bone marrow biopsy from these patients [6]. The main differential diagnoses for an leukemoid reaction (LR) include chronic myeloid leukemia (CML), chronic myelomonocytic leukemia (CMML), and, rarely, chronic neutrophilic leukemia (CNL). To reach an accurate diagnosis, it is essential to distinguish the LR from an MPN. Hence, pathologists not only have to report the absence of hematological malignancy but also alert the physician of the possibility of an occult CST. Most PLRs are neutrophilic predominant, although eosinophilic PLRs or mixed neutrophilic-eosinophilic PLRs were also previously found [6].

The diagnosis of LR over CML or CMML involves the absence of earlier granulocytic precursors, basophilia, or monocytosis, along with the features of reactive granulocytosis [6]. Also, contrary to LR, CML usually has a distinct myelocyte peak. A leukocyte alkaline phosphatase (LAP) score tends to be low in CML and high in PLR and CNL, however, the LAP score is rarely used since the widely available BCR/ABL1 assays can be obtained from peripheral blood. Contrary to LRs, the neutrophilia in CNL features a lack of left shift [6]. In our patient, BCR/ABL, JAK2V617, and CALR mutations were not detected.

Bone marrow examination in patients with PLR is usually hypercellular with myeloid hyperplasia, without an increase in blast percentage, and left-shifted myelopoiesis with toxic granulation [3]. In our patient, bone marrow biopsy showed hypercellular marrow and metastatic carcinoma involving 15% core cellularity.

Eosinophilia is defined as an increase in peripheral blood eosinophils, an absolute eosinophil count (AEC) of >500 cells/μL, and white blood cells (WBCs) that originate from the granulocytic lineage [2,3]. The incidence of eosinophilia in malignant tumors is about 1%. Hypereosinophilia (HES) - a more severe form of eosinophilia - is defined as AEC >1500 cells/μL with secondary organ damage [2].

The shift from asymptomatic to life-threatening paraneoplastic eosinophilia is rapid and difficult to diagnose, especially in lung cancer patients who are expected to have respiratory complications [7]. Our patient developed progressive respiratory failure warranted an aggressive chemotherapy regimen due to the burden of disease.

Although patients with paraneoplastic eosinophilia are usually asymptomatic, an elevated eosinophil count may be linked with dyspnea and wheezing [7]. Eosinophilia normally resolves with cancer-directed therapies. Matsumoto et al. reported chemotherapy results in a return to normal hematologic status [8]. Pandit et al. showed that tumor removal caused normalized leukocytosis and eosinophilia, and Anagnostopoulos et al. suggested that the return of eosinophilia may indicate tumor recurrence [9,10]. Tumor-related leukocytosis and paraneoplastic eosinophilia are not only prognostic signs but reflect the disease activity and treatment effectiveness [11].

There are several theories involving the mechanism of increased eosinophilia in solid tumors, including extensive dissemination of cancer in the bone marrow, local stimulation of the adjacent tissue to the tumor, tumor cells releasing eosinophilic chemotactic factors, metastatic tumor cells fueling the bone marrow causing eosinophil production, and tumor cell eosinophilactic factors activating eosinophil production by bone marrow cells and direct cytokine production [2,3,12].

The most widely accepted explanation is that the bone marrow, stimulated by release factors such as IL-5, stimulates eosinophil production [2,12]. There are many causes of eosinophilia in the peripheral blood, including leukemic processes, allergic disorders, and parasitic infections [12]. Although reports of increased eosinophils in the peripheral blood were previously described in solid tumor malignancies, it is still considered a rare event.

BRAF mutations are found in 50% of melanoma cases but only 1-4% of NSCLC cases. All somatic mutations in the BRAF gene are seen in the G-loop’s glycines in exon 11 or exon 15’s activation segment. BRAF mutations are commonly seen in hairy cell leukemia (100%), malignant melanoma (>60%), and papillary thyroid cancer (>50%) [4,13].

BRAF-mutant NSCLC usually shields the V600E allele (∼55%), causing constitutive activation of B-raf kinase and its downstream target extracellular regulated mitogen-activated protein (MAP) kinase (ERK)-9. The clinical development of BRAF V600E specific small inhibitors was established in melanoma, with two drugs (vemurafenib and dabrafenib) approved for the treatment of advanced disease. The BRAF gene encodes the serine and threonine-protein kinase BRAF, thereby regulating normal cell growth and spread [10]. Other frequent activating BRAF variants include G469A (∼35%) and D594G (∼10%) [4,13].

The mainstay of therapy for PLR is treating the underlying malignancy. The most effective ways to decrease WBC counts in responsive tumors are surgical resection, radiotherapy, and chemotherapeutic agents [6]. Lung cancer patients presenting with abnormally high counts of eosinophils should be treated with a combination of corticosteroids, hydroxyurea, and anticancer drugs. Corticosteroids and hydroxyurea enhance the benefits of anticancer treatment and prevent the development of aggressive and life-threatening eosinophilia, as seen in our patient [7].

Patients with melanoma have significantly prolonged survival with combined MEK and BRAF inhibition as compared to BRAF-monotherapy. Preclinical lung cancer models displayed that combination therapy was also more active than single agent [13].

Eosinophilia is usually treated with corticosteroids [7]. For paraneoplastic eosinophilia, there is no specific treatment other than treating the underlying malignancy. Unlike primary eosinophilia which response to steroids, paraneoplastic eosinophilia can be resistant. Sometimes response rates can be increased with the combination of steroids and hydroxyurea. However, management is mainly based on treating the malignancy by chemotherapy or surgery [7]. 

The presence of paraneoplastic eosinophilia and linked with a poor prognosis and more aggressive malignancies. Since this condition is rare, an extensive workup is needed to identify the cause and initiate treatment urgently. Furthermore, it is important to identify early signs of aggressive paraneoplastic eosinophilia to initiate corticoste­roid treatment prior to end-organ failure [7].

## Conclusions

This is a rare case of a patient with metastatic NSCLC and a BRAF V600E mutation, who presented with hypoxic respiratory insufficiency and extreme neutrophilia and eosinophilia as a hematologic paraneoplastic syndrome and responded to targeted therapy with combined MEK and BRAF inhibitors. Corticosteroids and hydroxyurea enhance the benefits of anticancer treatment and prevent the development of aggressive and life-threatening eosinophilia, as seen in our patient.
